# Measurement Compensation for Time-Delay Seeker and Three-Dimensional Adaptive Guidance Law Design

**DOI:** 10.3390/s21123977

**Published:** 2021-06-09

**Authors:** Yukuan Liu, Guanglin He, Zenghui Qiao, Zhaoxuan Guo, Zehu Wang

**Affiliations:** 1 Science and Technology on Electromechanical Dynamic Control Laboratory, Beijing Institute of Technology, Beijing 100081, China; 3120185132@bit.edu.cn (Y.L.); 3120190185@bit.edu.cn (Z.G.); 3120200250@bit.edu.cn (Z.W.); 2 PLA Strategic Support Force, Beijing 100000, China; qiaozenghui81@163.com

**Keywords:** measurement compensation, time-delay system, predictive active disturbance rejection control, guidance law, sliding mode control, adaptive super-twisting

## Abstract

The time delay of seekers has grown to be a serious issue for tactical missile guidance with the development of flight vehicle technologies. To address the problem, a measurement compensation system for the seeker, with lags and delays based on predictive active disturbance rejection control, is proposed. In addition, to eliminate the effects of target maneuvers to the tactical missile guidance, an adaptive finite-time convergent sliding mode guidance law, based on super-twisting algorithm, is proposed in three-dimensional missile-target engagement kinematics. Specifically, the compensation system consists of a predictive tracking structure and an active disturbance rejection control system, which could follow a virtual measurement without lags and delays. The compensation system has advantages in disturbance rejection and model inaccuracy addressing, compared with existing compensation methods for seeker measurement. As for the sliding mode guidance law design, the proposed approach is based on an improved super-twisting algorithm with fast convergent adaptive gains, which has advantages in addressing unknown but bounded target maneuvers and avoiding chattering of the classical sliding mode control. As a result, the measurement compensation system and the adaptive sliding mode guidance law is verified robust and effective under the proposed constraints by the simulation examples.

## 1. Introduction

For tactical missiles, seeker (or detector) measurement and precision guidance are two key technologies, which would influence homing performance significantly. In practice, due to the seeker and filter dynamics, the complex information processing method and the target detection algorithm, significant seeker lags and delays always exist in the missile system. Consequently, the lags and delays may lead to the degradation of the guidance precision or even missing targets, as studied in [1].

This problem motivates researchers to develop data compensation methods for the time-delay seeker. The general goal of these methods is to estimate the ideal measurement utilizing the undesirable output. Some researchers assumed the seeker dynamics model as a first-order lag system [2,3,4,5], and achieved adequate outcomes for many purposes. However, in many real situations, there exists both dynamics lags and pure time delays in the seeker system [6,7]. In the literatures considering this problem, the compensation or estimation approaches can be broadly divided into two directions:

1. Approaches based on a Kalman filter [8,9,10,11,12];
2. Approaches based on a predictor observer [13].

The former type of approach aims to obtain the estimation of time delays by fusing the system state variable and the measurement variable. Reference [8] developed a Kalman filter insensitive to modeling errors to compensate one-sample delay in arrival of line-of-sight angle measurements. Additionally, [9,10], also based on Kalman filters, proposed feedback structures to model and estimate the delayed and lost measurements in the guidance systems, employing command to line-of-sight strategy. In [11], a seeker time-delay model and a filter for obtaining the look angle rate in the feedback signal loop, based on the concept of the model matching technique, were introduced. Similarly, [12] suggested a novel Kalman filter dynamic for time-delayed and noisy measurements of optical sensors, and analyzed the robustness of the delayed pointing error measurements. In general, these approaches build simple feedback loops and utilize filters to estimate the delayed state variables. However, these methods require an accurate delayed seeker model or target engagement model, which are always inaccurate in practice. Consequently, these approaches might have poor effectiveness and robustness under the uncertain situations.

For the second strategy, in [13], a classical predictor observer was introduced, which had considerable engineering significance for linear time-delayed seeker systems. The proposed predictor observer consists of two components: a classical predictor feedback and a Luenberger observer. Though the proposed observer can also be implemented as a Kalman filter result from the identical structure, this approach was the first step in studying the application of predictor feedback to a time-delay missile guidance system. However, there is also much room for improvement. The predictor observer still has a strong requirement for accurate system models, including exact seeker delays. Additionally, the structure of the predictor leads to a conflict between increasing tracking speed and improving disturbances rejection.

In the engineering domain, there are other advanced approaches addressing the time-delay problem. In [14,15], cascade observers were employed for output-feedback control under parametric uncertainties, disturbances, and arbitrary sensor delays. On the basis of a predictor and sliding mode control, [16] proposed a sliding mode predictive control for linear uncertain systems with time delays. Reference [17] utilized disturbance observers to design a prediction-based control for multi-area interconnected power systems with input time delays. In addition, many time-delay compensation control strategies implementing active disturbance rejection control (ADRC) have been studied [18,19,20,21,22,23]. References [19,20] proposed modified ADRC structures by adding a time-delay loop and reconstructing a Smith predictor, respectively. Combined with a Smith predictor, [21] proposed a fast self-learning ADRC algorithm, while [22] transferred the system into a two-degree-freedom feedback control structure and used an internal model control for the delayed processes. Moreover, [23] developed a novel higher-order ADRC method with a selectable response smoothing degree based on integrator-plus-dead-time models, which give better results in time-delayed compensation than simpler solutions [19,20]. Overall, the predictive ADRC method has adequate performances and properties in addressing time-delay system and rejecting disturbances, and it shows strong robustness under uncertain system modelling. However, this approach has not been applied in the problem of tactical missile guidance with time-delay seeker.

As for the development of guidance laws, sliding mode control (SMC) theory has been regarded as a powerful tool to design guidance laws with constraints. By constructing the sliding manifold with the first-order states (LOS angle) and second-order states (LOS angular rate), SMC method can reach global stabilization, where the sliding mode surface converges to zero [24,25]. In [26], a nonsingular terminal sliding mode guidance law was implemented, which considered the impact angle constraint. Reference [27] proposed an adaptive guidance law for obtaining a specified impact angle, and applied into a hypersonic vehicle. Adding a second-order sliding mode observer, [28] proposed a robust guidance law with autopilot lag consideration. Reference [29] presented an integral sliding mode guidance law which could resolve the steady-state error problem of the traditional SMC. An optimization design with the neural network was designed to improve the fuzzy variable structure of sliding mode in [30]. However, the discontinuity of sliding mode controllers may cause an undesirable chattering of the system with fast actuators. Furthermore, the SMC guidance laws require certain target maneuvers, which are always unavailable in practice.

Thus, the adaptive super-twisting algorithm (STA) [31] controller became popular due to its excellent property of eliminating chattering and disturbances. The main goal of adaptive controller design is to ensure a dynamical adaption of the control gains in order to be as small as possible while still sufficient to counteract the disturbances and ensure a sliding mode [32,33,34]. In [35], an adaptive STA guidance law which could converge in finite time was designed based on the increasing STA gains, as proposed in [32]. There is a distinct disadvantage in that the gain will not decrease; as a consequence, the controller will not follow the disturbance when it is decreasing. To address this problem, reference [36] utilized an equivalent control strategy, as proposed in [33], and designed a STA-like guidance law with actuator faults constraint. Considering the target maneuvering as system disturbance, the guidance laws based on STA control have a smooth output without obtaining target maneuvering. However, due to the linear error form between the adaptive gain and the disturbance [33,36], the convergence speed of the error is slow and results in slow, even false, adaptive gains.

Inspired by above works, this study proposes a measurement compensation method based on predictive ADRC for time-delay seeker and a three-dimensional adaptive guidance law based on SMC and adaptive STA control. The main contributions of this paper can be concluded as follows:

1. A predictive ADRC method is first introduced into the tactical missile system to compensate the seeker lags and delays, which can achieve satisfied results under the approximate delay assumption and noisy measurement;
2. In order to design the sliding mode guidance law, a modified adaptive STA controller is applied to the tactical system to obtain adaptive gains with a faster convergence error form, and the stability of the control system is also analyzed.

The rest of the paper is organized as follows: Section 2 states the missile-target engagement kinematics and preliminaries; Section 3 builds a time-delay seeker measurement compensation system based on predictive ADRC; Section 4 designs a sliding mode guidance law based on the compensation measurement and adaptive STA control; Section 5 discusses the effectiveness of the proposed methods, utilizing several simulation examples.

## 2. Problem Statement

In this section, kinematics model of missile-target guidance system and first-order seeker lag during the engagement phase are presented for the later guidance design law design. Moreover, some preliminaries are also considered for further application to facilitate the design.

### 2.1. Missile-Target Engagement Kinematics

**Figure 1:**
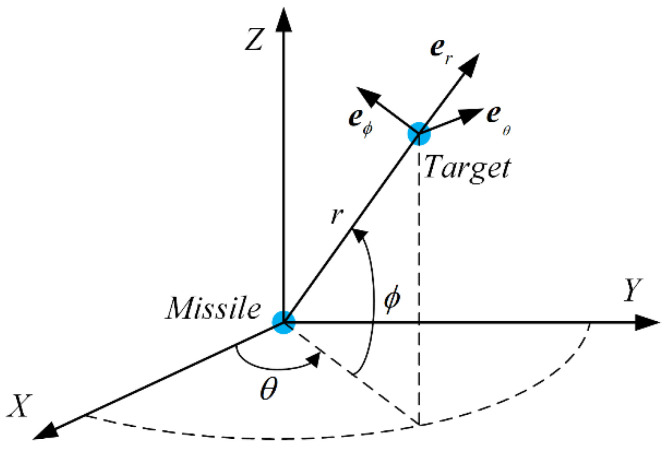
Three-dimension schematic diagram of missile-target engagement.

As illustrated in Figure 1, the three-dimensional pursuit geometry relationship between missile and target in inertial coordinate system (X,Y,Z). With two-times rotation of the inertial coordinate, the line-of-sight (LOS) coordinate system (r,θ,ϕ) could be obtained, where r, θ and ϕ are the relative distance between the point masses of missile and target, the azimuth, and elevation angles of the LOS, respectively. The process of the rotation could be formulated as

(1) $$\left[\begin{array}{c} e_{r}\\ e_{\theta }\\ e_{\phi } \end{array}\right]=L\left(\phi ,\theta \right)\left[\begin{array}{c} X\\ Y\\ Z \end{array}\right]$$

where ${\left[\begin{array}{ccc} e_{r} & e_{\theta } & e_{\phi } \end{array}\right]}^{T}$ is the component of the unit vectors of the LOS coordinate system, and L(ϕ,θ) is the two-times rotation matrix, which is given by

(2) $$L\left(\phi ,\theta \right)=\left[\begin{array}{ccc} \cos\theta \cos\phi & \cos\phi \sin\theta & \sin\phi \\ -\sin\theta & \cos\theta & 0\\ -\sin\phi \cos\theta & -\sin\theta \sin\phi & \cos\phi \end{array}\right]$$

The relative velocity in LOS coordinate system ***V*** satisfies the following differential form

(3) $$\frac{dV}{dt}=\frac{\delta V}{\delta t}+\omega \times V=a_{T}-a_{M}$$

where $V={\left[\begin{array}{ccc} \dot{r} & r\dot{\theta }\cos\phi & r\dot{\phi } \end{array}\right]}^{T}$; dV/dt and δV/δt represent the absolute derivative of V in inertial coordinate system, and the relative derivative of V in LOS coordinate system, respectively; $a_{T}$ and $a_{M}$ represent the accelerations of the target and missile, respectively; and ω represents the relative rotation angular velocity of LOS coordinate system relative to inertial coordinate, which is given by

(4) $$\omega =L(\phi ,\theta )\left[\begin{array}{c} 0\\ 0\\ \dot{\theta } \end{array}\right]+\left[\begin{array}{c} 0\\ -\dot{\phi }\\ 0 \end{array}\right]=\left[\begin{array}{c} \dot{\theta }\sin\phi \\ -\dot{\phi }\\ \dot{\theta }\cos\phi \end{array}\right]$$

Substituting Equations (2) and (4) into the Equation (3), the three-dimension missile-target engagement kinematic could be described as

(5) $$\begin{array}{l} \ddot{r}={\dot{\theta }}^{2}\cos^{2}\phi r+{\dot{\phi }}^{2}r+a_{Tr}-a_{Mr}\\ \ddot{\theta }=2\dot{\phi }\dot{\theta }\tan\phi -\frac{2\dot{r}\dot{\theta }}{r}+\frac{a_{T\theta }}{r\cos\phi }-\frac{a_{M\theta }}{r\cos\phi }\\ \ddot{\phi }=-{\dot{\theta }}^{2}\sin\phi \cos\phi -\frac{2\dot{r}\dot{\phi }}{r}+\frac{a_{T\phi }}{r}-\frac{a_{M\phi }}{r} \end{array}$$

**Remark** **1.** *From Equation (5), it can be observed that r=0 and ϕ=±π/2 are singular points. However, due to the physical shapes of the missile and target in real practice, the relative distance converges in a neighborhood of zero, which means r=0 does not occur throughout the detection and guidance process. In addition, regular points ϕ=±π/2 have been proven unstable in [26]. Hence, the control system just crosses these points without stay.*

During the design of guidance law, desired LOS terminal angles $\theta_{d}$ and $\phi_{d}$ are always considered to be the constraint. Denoting $x_{1}={\left[\begin{array}{cc} x_{1\theta } & x_{1\phi } \end{array}\right]}^{T}={\left[\begin{array}{cc} \theta -\theta_{d} & \phi -\phi_{d} \end{array}\right]}^{T}$ and $x_{2}={\dot{x}}_{1}={\left[\begin{array}{cc} x_{2\theta } & x_{2\phi } \end{array}\right]}^{T}={\left[\begin{array}{cc} \dot{\theta } & \dot{\phi } \end{array}\right]}^{T}$ as the control variables, Equation (5) can be rewritten as

(6) $$\begin{array}{l} {\dot{x}}_{1}=x_{2}\\ {\dot{x}}_{2}=F(x)+Bu+\Delta \end{array}$$

where

(7) $$\begin{array}{c} F=\left[\begin{array}{c} 2x_{2\theta }x_{2\phi }\tan\phi -\frac{2\dot{r}x_{2\theta }}{r}\\ -x_{2\theta }^{2}\sin\phi \cos\phi -\frac{2\dot{r}x_{2\phi }}{r} \end{array}\right],\;B=\left[\begin{array}{cc} -\frac{1}{r\cos\phi } & 0\\ 0 & -\frac{1}{r} \end{array}\right]\\ u=a_{M}={\left[\begin{array}{cc} a_{M\theta } & a_{M\phi } \end{array}\right]}^{T},\;\Delta =\left[\begin{array}{cc} \Delta_{1} & \Delta_{2} \end{array}\right]={\left[\begin{array}{cc} \frac{a_{T\theta }}{r\cos\phi } & \frac{a_{T\phi }}{r} \end{array}\right]}^{T} \end{array}$$

### 2.2. Preliminaries

**Notation** **1.** *For any given vector $x={\left[\begin{array}{cccc} x_{1} & x_{2} & \cdots & x_{n} \end{array}\right]}^{T}$, denote its absolute value as $\left|x\right|={\left[\begin{array}{cccc} \left|x_{1}\right| & \left|x_{2}\right| & \cdots & \left|x_{n}\right| \end{array}\right]}^{T}$, its time derivative as $\dot{x}={\left[\begin{array}{cccc} {\dot{x}}_{1} & {\dot{x}}_{2} & \cdots & {\dot{x}}_{n} \end{array}\right]}^{T}$, its sign function as $sign(x)={\left[sign(\begin{array}{cccc} x_{1}) & sign(x_{2}) & \cdots & sign(x_{n}) \end{array}\right]}^{T}$, its second power as $x^{2}={\left[\begin{array}{cccc} x_{1}^{2} & x_{2}^{2} & \cdots & x_{n}^{2} \end{array}\right]}^{T}$, and its 2-norm as $\left\|x\right\|=\sqrt{x^{T}x}$. For any positive definite matrix P, denote $\lambda_{\min}(P)$ and $\lambda_{\max}(P)$ to represent the minimum and maximum eigenvalues of P, respectively, and satisfying following inequation: $\lambda_{\min}(P){\left\|x\right\|}^{2}\leq x^{T}Px\leq \lambda_{\max}(P){\left\|x\right\|}^{2}$. In addition, denote operation symbol “∘” as the Hadamard product symbol, denote operation symbol “⊗” as the Kronecker product symbol.*

**Assumption** **1.** *Assume the target accelerations $a_{T\theta }$ and $a_{T\phi }$ are unknown but bounded, continuous, and differentiable. Additionally, Δ is unknown but bounded, continuous, and differentiable and $\dot{\Delta }={\left[\begin{array}{cc} {\dot{\Delta }}_{1} & {\dot{\Delta }}_{2} \end{array}\right]}^{T}$ is unknown but bounded and continuous. Assume the absolute values of Δ and $\dot{\Delta }$ satisfy following inequation*

(8) $$\left|\Delta_{i}\right|\leq \Delta_{\max},\;\left|{\dot{\Delta }}_{i}\right|\leq {\dot{\Delta }}_{\max},\;i=1,2$$

*where $\Delta_{\max}$ and ${\dot{\Delta }}_{\max}$ are positive constants.*

**Assumption** **2.** *The seeker time delay τ is an approximate known positive constant.*

**Lemma** **1.** *For a first-order nonlinear system $\dot{x}=f(x,t)$, $x\in {\mathbb{R}}^{n}$. Assume there exists continuous and positive definite function V(x) satisfies the following inequality [37]*

(9) $$\dot{V}(x)+\lambda_{1}V(x)+\lambda_{2}V^{\lambda_{3}}(x)\leq 0$$

*where $\lambda_{1},\lambda_{2}>0$, and $\lambda_{3}\in (0,1)$ are constants. Then, there exists a region $U_{0}\in {\mathbb{R}}^{n}$ such that any V(x) starting from this region can reach V(x)≡0 in a finite time $T_{r}$, which is formulated as*

(10) $$T_{r}\leq \frac{1}{\lambda_{1}(1-\lambda_{3})}\ln\left(1+\frac{\lambda_{1}}{\lambda_{2}}V^{1-\lambda_{3}}(x_{0})\right)$$

*where $V(x_{0})$ is the initial value of V(x).*

## 3. Measurement Compensation System for Time-Delay Seeker Based on Predictive ADRC

In this section, a seeker measurement model with lags and delays is formulated. Following this, based on predictive ADRC theory, a measurement compensation system is reconstructed, and the calculation process is given.

### 3.1. Modelling for Time-Delay Seeker Considering Lag Dynamics

**Figure 2:**
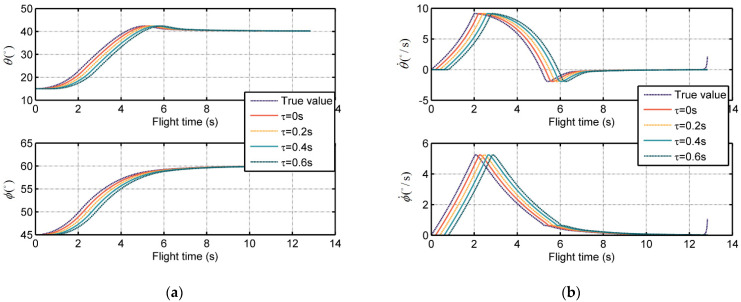
The seeker measurements under different time delays: (**a**) The measured LOS angles with lags and delays; (**b**) The measured LOS angular rates with lags and delays.

In the process of homing missile guidance, the guidance loop needs to obtain the real-time LOS angles and LOS angular rates. Denoting $\theta_{g}$ and $\phi_{g}$ are the measured azimuth and elevation angles of the seeker, respectively. Then, considering first-order seeker dynamics and time delay, $\theta_{g}$ and $\phi_{g}$ can be formulated as

(11) $$\begin{array}{l} {\dot{\theta }}_{g}=-\frac{1}{T_{s}}\theta_{g}+\frac{1}{T_{s}}\theta \left(t-\tau \right)\\ {\dot{\phi }}_{g}=-\frac{1}{T_{s}}\phi_{g}+\frac{1}{T_{s}}\phi \left(t-\tau \right) \end{array}$$

where $T_{s}$ is the time scale of the seeker dynamics and τ is the pure time delay of the seeker, which is an unknown but positive constant. Based on Equation (11), Figure 2a,b show the trend of LOS angles and LOS angular rates measurement, respectively, in respect to the flight time of missiles under different pure time delays.

For Equation (11), denoting $x_{3}={\left[\begin{array}{cc} x_{31} & x_{32} \end{array}\right]}^{T}={\left[\begin{array}{cc} \theta_{g} & \phi_{g} \end{array}\right]}^{T}$, $y_{3}={\left[\begin{array}{cc} y_{31} & y_{32} \end{array}\right]}^{T}=x_{3}$, and $u_{3}={\left[\begin{array}{cc} u_{31} & u_{32} \end{array}\right]}^{T}={\left[\begin{array}{cc} \theta \left(t\right) & \phi \left(t\right) \end{array}\right]}^{T}$, rewrite it as

(12) $$\begin{array}{l} {\dot{x}}_{3}=-\frac{1}{T_{s}}x_{3}+\frac{1}{T_{s}}u_{3}\left(t-\tau \right)\\ y_{3}=x_{3} \end{array}$$

From Equation (12), one can observe that the input variable $u_{3}$ is the LOS angle without time delay and dynamics lag, which is ideal for the missile guidance system. To estimate $u_{3}$ and reconstruct the seeker measurements, the following analysis is based on S-domain for convenience. Denoting s as the complex variable, the S-domain transfer function of system (12) can be formulated as

(13) $$\frac{y_{3}}{u_{3}}=\frac{1}{T_{s}+1}e^{-\tau s}$$

Introduce a virtual output variable ${\bar{y}}_{3}$ which is given by

(14) $${\bar{y}}_{3}=\frac{1}{T_{s}+1}u_{3}$$

It is obvious that ${\bar{y}}_{3}$ has no time delay, implying that if we could track its value we could eliminate the effects of time delay. Inspired by [18], this paper proposes a novel measurement reconstruction system based on tracking differentiator and active disturbance rejection control theory to track ${\bar{y}}_{3}$ and reject disturbance.

### 3.2. Measurement Compensation System Design

**Figure 3:**
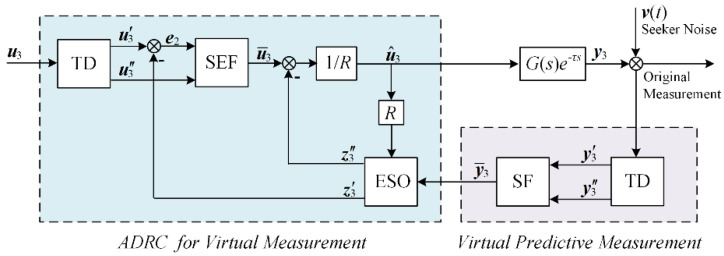
The ADRC compensation structure for seeker.

Figure 3 shows the entire measurement reconstruction structure for the seeker. The reconstruction process can be divided into two parts:

1. Predictive tracking for virtual measurement ${\bar{y}}_{3}$

This part is formed from an optimal tracking differentiator (TD) and a state feedback (SF) controller. The discrete TD equation can be formulated as

(15) $$\left\{ \begin{array}{l} fy\left(k\right)=fhan\left(y_{3}^{\prime }\left(k\right)-y_{3}\left(k\right),y_{3}^{\prime \prime }\left(k\right),p_{0},h_{0}\right)\\ y_{3}^{\prime }\left(k+1\right)=y_{3}^{\prime }\left(k\right)+hy_{3}^{\prime \prime }\left(k\right)\\ y_{3}^{\prime \prime }\left(k+1\right)=y_{3}^{\prime \prime }\left(k\right)+hfy\left(k\right) \end{array}\right.$$

where k and k+1 are previous time mark and current time mark, respectively; h is sample time; $p_{0}$ and $h_{0}$ are designed positive constants, higher $p_{0}$ leads to higher tracking speed, and higher $h_{0}$ leads to better filtering effect to the system; $y_{3}^{\prime }={\left[\begin{array}{cc} y_{31}^{\prime } & y_{32}^{\prime } \end{array}\right]}^{T}$ and $y_{3}^{\prime \prime }={\left[\begin{array}{cc} y_{31}^{\prime \prime } & y_{32}^{\prime \prime } \end{array}\right]}^{T}$ are the output of TD, which tracks ${\bar{y}}_{3}$ and ${\dot{\bar{y}}}_{3}$, respectively; and function $fhan\left(y_{3}^{\prime }-y_{3},y_{3}^{\prime \prime },p_{0},h_{0}\right)$ is given by

(16) $$\begin{array}{cc} \left\{ \begin{array}{l} g=p_{0}h_{0}\\ g_{0}=h_{0}g\\ m_{i}=(y_{3i}^{\prime }-y_{3i})+h_{0}y_{3i}^{\prime \prime }\\ a_{0i}=\sqrt{g^{2}+8p_{0}\left|m_{i}\right|}\\ a_{i}=\{\begin{array}{cc} y_{3i}^{\prime \prime }+(a_{0i}-g)sign(m_{i})/2, & \left|m_{i}\right|>g_{0}\\ y_{3i}^{\prime \prime }+m_{i}/h_{0} & \left|m_{i}\right|\leq g_{0} \end{array}\\ fhan_{i}=\{\begin{array}{cc} -p_{0}sign(a_{i}), & \left|a_{i}\right|>g\\ -p_{0}a_{i}/g & \left|a_{i}\right|\leq g \end{array} \end{array}\right. & \left(i=1,2\right) \end{array}$$

It can be concluded that TD in the form of Equation (15) has the property to filter noises due to its first-order differentiation loop. Then the SF controller in a classical predictor observer can be described as

$${\bar{y}}_{3}\left(k+1\right)=y_{3}^{\prime }\left(k\right)+\tau y_{3}^{\prime \prime }\left(k\right)$$

However, τ is an approximate known constant based on Assumption 2. Thus, rewrite above equation as

(17) $${\bar{y}}_{3}\left(k+1\right)=y_{3}^{\prime }\left(k\right)+\alpha \bar{\tau }y_{3}^{\prime \prime }\left(k\right)$$

where 0<α<1 and $\bar{\tau }>0$ are designed constants.

2. ADRC for virtual measurement

In the process of predictive tracking for virtual measurement ${\bar{y}}_{3}$, approximate delay parameters are used, which add uncertainty to the system. In addition, noises and lags occur in the seeker detection process, and disturbances also exist when ${\bar{y}}_{3}$ has dynamic changes. To compensate lag, and reject the uncertainty and disturbances including noises, an ADRC system is introduced, which consists of three subsystems: TD, extended state observer (ESO), and state error feedback (SEF).

- Transient process based on TD

The TD is utilized to track $u_{3}$ and ${\dot{u}}_{3}$, which is given by

(18) $$\left\{ \begin{array}{l} fu\left(k\right)=fhan\left(u_{3}^{\prime }\left(k\right)-u_{3}\left(k\right),u_{3}^{\prime \prime }\left(k\right),p_{0},h_{0}\right)\\ u_{3}^{\prime }\left(k+1\right)=u_{3}^{\prime }\left(k\right)+hu_{3}^{\prime \prime }\left(k\right)\\ u_{3}^{\prime \prime }\left(k+1\right)=u_{3}^{\prime \prime }\left(k\right)+hfu\left(k\right) \end{array}\right.$$

where $u_{3}^{\prime }={\left[\begin{array}{cc} u_{31}^{\prime } & u_{32}^{\prime } \end{array}\right]}^{T}$ and $u_{3}^{\prime \prime }={\left[\begin{array}{cc} u_{31}^{\prime \prime } & u_{32}^{\prime \prime } \end{array}\right]}^{T}$ are the output of TD, which tracks $u_{3}$ and ${\dot{u}}_{3}$, respectively; function *fhan* is given in Equation (16).

- State and disturbance estimation based on ESO

The ESO is used to eliminate disturbances and estimate the real value of ${\bar{y}}_{3}$, which is formulated as

(19) $$\left\{ \begin{array}{l} e_{1}\left(k\right)=z_{3}^{\prime }\left(k\right)-{\bar{y}}_{3}\left(k\right)\\ z_{3}^{\prime }\left(k+1\right)=z_{3}^{\prime }\left(k\right)+h\left(z_{3}^{\prime \prime }\left(k\right)+R{\hat{u}}_{3}\left(k\right)\right)-\beta_{1}fal\left(e_{1}\left(k\right)\right)\\ z_{3}^{\prime \prime }\left(k+1\right)=z_{3}^{\prime \prime }\left(k\right)-\beta_{2}fal\left(e_{1}\left(k\right)\right) \end{array}\right.$$

where input gain R is a positive constant; error gains $\beta_{1}$ and $\beta_{2}$ are positive constants; ${\hat{u}}_{3}={\left[\begin{array}{cc} {\hat{u}}_{31} & {\hat{u}}_{32} \end{array}\right]}^{T}={\left[\begin{array}{cc} \hat{\theta } & \hat{\phi } \end{array}\right]}^{T}$ is the observed quantity of $u_{3}$; $e_{1}={\left[\begin{array}{cc} e_{11} & e_{12} \end{array}\right]}^{T}$ is the error between the estimated and real value of ${\bar{y}}_{3}$; $z_{3}^{\prime }={\left[\begin{array}{cc} z_{32}^{\prime } & z_{32}^{\prime } \end{array}\right]}^{T}$ and $z_{3}^{\prime \prime }={\left[\begin{array}{cc} z_{31}^{\prime \prime } & z_{32}^{\prime \prime } \end{array}\right]}^{T}$ are the output values of the ESO, which represent the estimation of ${\bar{y}}_{3}$ and error, respectively; and $fal\left(e_{1i}\right)$ is error function, which is given by

(20) $$\begin{array}{cc} fal\left(e_{1i}\right)=\{\begin{array}{cc} {\left|e_{1i}\right|}^{\mu }sign\left(e_{1i}\right) & \left|e_{1i}\right|>\delta \\ e_{1i}/\delta^{1-\mu } & \left|e_{1i}\right|\leq \delta \end{array} & \left(i=1,2\right) \end{array}$$

where 0<μ≤1 and δ>0 are constants, which represent order and boundary of $e_{1}$, respectively.

**Remark** **2.** *The stabilization of ESO systems has been proven in [18], which clarified that when the observer gains are selected appropriately, the errors of the ESO could converge to neighborhood of zero in finite time.*

- Input estimation based on SEF

The SEF can calculate the error between $u_{3}^{\prime }$ and $z_{3}^{\prime }$, which is given by

(21) $$\left\{ \begin{array}{l} e_{2}=u_{3}^{\prime }-z_{3}^{\prime }\\ {\bar{u}}_{3}=\beta_{3}e_{2} \end{array}\right.$$

where ${\bar{u}}_{3}={\left[\begin{array}{cc} {\bar{u}}_{31} & {\bar{u}}_{32} \end{array}\right]}^{T}$ is the error feedback; $\beta_{3}$ is positive constant. Thus, ${\hat{u}}_{3}$ can be formulated as

(22) $${\hat{u}}_{3}=\left({\bar{u}}_{3}-z_{3}^{\prime \prime }\right)/R$$

As a result, using Equations (15)–(22), the input estimation ${\hat{u}}_{3}$ can be calculated. Meanwhile, the delayed measurement is compensated, and the LOS angle is reconstructed during the process.

**Remark** **3.** *The reconstruction of LOS angular rate can be described by the difference of LOS angle, which is given by*

(23) $$\begin{array}{l} \hat{\dot{\theta }}\left(k+1\right)=\frac{\hat{\theta }\left(k+1\right)-\hat{\theta }\left(k\right)}{h}\\ \hat{\dot{\phi }}\left(k+1\right)=\frac{\hat{\phi }\left(k+1\right)-\hat{\phi }\left(k\right)}{h} \end{array}$$

*where $\hat{\dot{\theta }}\left(k+1\right)$ and $\hat{\dot{\phi }}\left(k+1\right)$ are the estimations of azimuth and elevation angular rates at current time, respectively.*

## 4. Adaptive Guidance Law Design Considering Terminal Angle Constraint

In this section, a fast convergent nonsingular sliding manifold variable is introduced. An adaptive sliding mode guidance law, regardless of target maneuvers and based on STA, is proposed, and the stabilization of the guidance law is studied based on Lyapunov theory.

### 4.1. Design of Adaptive Sliding Mode Guidance Law Based on STA

Considering both convergence speed and nonsingularity, the following sliding manifold for system (6) is proposed, which is given by

(24) $$\sigma =x_{2}+k_{1}x_{1}+k_{2}w(x_{1})$$

where $\sigma ={\left[\begin{array}{cc} \sigma_{1} & \sigma_{2} \end{array}\right]}^{T}$ is the sliding manifold vector; $k_{1}$ and $k_{2}$ are positive constants; and term $w(x_{1})={\left[\begin{array}{cc} w(x_{11}) & w(x_{12}) \end{array}\right]}^{T}$ is formulated as

(25) $$w(x_{1i})=\begin{array}{cc} \{\begin{array}{cc} {\left|x_{1i}\right|}^{k_{3}}sign\left(x_{1i}\right), & \sigma_{i}=0\;or\;{\bar{\sigma }}_{i}\neq 0,\left|x_{1i}\right|\geq \xi \\ k_{4}x_{1i}+k_{5}x_{1i}^{2}sign\left(x_{1i}\right), & {\bar{\sigma }}_{i}\neq 0,\left|x_{1i}\right|<\xi \end{array} & \left(i=1,2\right) \end{array}$$

where ${\bar{\sigma }}_{i}=x_{2i}+k_{1}x_{1i}+k_{2}{\left|x_{1i}\right|}^{k_{3}}sign\left(x_{1i}\right)$; constants $0<k_{3}<1$, ξ>0; $k_{4}$ and $k_{5}$ are designed to keep $w(x_{1i})$ continuous, which satisfies

(26) $$k_{4}=\left(2-k_{3}\right)\varpi^{k_{3}-1},\;k_{5}=\left(2-k_{3}\right)\varpi^{k_{3}-2}$$

Then the time derivative of σ can be formulated as

(27) $$\dot{\sigma }={\dot{x}}_{2}+k_{1}x_{2}+k_{2}\dot{w}(x_{1})$$

where term $\dot{w}(x_{1})$ is given by

(28) $$\dot{w}(x_{1i})=\begin{array}{cc} \{\begin{array}{cc} k_{3}x_{1i}^{k_{3}-1}x_{2i}, & \sigma_{i}=0\;or\;{\bar{\sigma }}_{i}\neq 0,\left|x_{1i}\right|\geq \xi \\ k_{4}x_{2i}+2k_{5}x_{1i}x_{2i}, & {\bar{\sigma }}_{i}\neq 0,\left|x_{1i}\right|<\xi \end{array} & \left(i=1,2\right) \end{array}$$

According to Equations (15)–(23), denote

(29) $${\hat{x}}_{1}={\left[\begin{array}{cc} {\hat{x}}_{1\theta } & {\hat{x}}_{1\phi } \end{array}\right]}^{T}={\left[\begin{array}{cc} \hat{\theta }-\theta_{d} & \hat{\phi }-\phi_{d} \end{array}\right]}^{T},\;{\hat{x}}_{2}={\left[\begin{array}{cc} {\hat{x}}_{2\theta } & {\hat{x}}_{2\phi } \end{array}\right]}^{T}={\left[\begin{array}{cc} \hat{\dot{\theta }} & \hat{\dot{\phi }} \end{array}\right]}^{T}$$

Then Substituting Equation (6) into Equation (27) and replacing ϕ, $x_{1}$ and $x_{2}$ with $\hat{\phi }$, ${\hat{x}}_{1}$ and ${\hat{x}}_{2}$ yields

(30) $$\dot{\sigma }=\bar{F}(\hat{x})+Ba_{M}+k_{1}{\hat{x}}_{2}+k_{2}\dot{w}({\hat{x}}_{1})+\Delta$$

where

(31) $$\bar{F}(\hat{x})=\left[\begin{array}{c} 2{\hat{x}}_{2\theta }{\hat{x}}_{2\phi }\tan\hat{\phi }-\frac{2\dot{r}{\hat{x}}_{2\theta }}{r}\\ -{\hat{x}}_{2\theta }^{2}\sin\hat{\phi }\cos\hat{\phi }-\frac{2\dot{r}{\hat{x}}_{2\phi }}{r} \end{array}\right]$$

In order to eliminate the chattering of the sliding mode control and ensure the effectiveness of the guidance law facing maneuvering target. The adaptive STA control method is utilized in this paper. On the bases of adaptive super-twisting control theory and the nonlinear sliding manifold, regarding Δ as the system disturbance, a target-independent guidance law with adaptive gains based on STA can be formulated as

(32) $$\begin{array}{l} a_{M}=-B^{-1}(\bar{F}(\hat{x})+k_{1}{\hat{x}}_{2}+k_{2}\dot{w}({\hat{x}}_{1})-\eta (t))\\ \eta (t)=-a_{1}(t)\circ \frac{\sigma }{{\left\|\sigma \right\|}^{1/2}}-a_{2}(t)\circ \sigma +y(t)\\ \dot{y}(t)=-a_{3}(t)\circ \frac{\sigma }{\left\|\sigma \right\|}-a_{4}(t)\circ \sigma \end{array}$$

where

(33) $$\begin{array}{cc} \begin{array}{l} a_{1}(t)=b_{1}\sqrt{L(t)},\\ a_{3}(t)=b_{3}L(t), \end{array} & \begin{array}{l} a_{2}(t)=b_{2}L(t)\\ a_{4}(t)=b_{4}L^{2}(t) \end{array} \end{array}$$

where $b_{1}$, $b_{2}$, $b_{3}$, and $b_{4}$ are positive constants. In reference [33], to compensate the objective system uncertainty, an equivalent control variable ${\bar{u}}_{eq}(t)$ and its error variable δ(t) are introduced, which satisfies

(34) $$\begin{array}{l} {\dot{\bar{u}}}_{eq}(t)=\frac{1}{\upsilon }\left(a_{3}(t)\circ \frac{\sigma }{\left\|\sigma \right\|}-{\bar{u}}_{eq}(t)\right)\\ \delta (t)=L\left(t\right)-\frac{1}{\varpi b_{3}}\left|{\bar{u}}_{eq}(t)\right|-\varepsilon \end{array}$$

where ϖ is a constant, satisfies 0<ϖ<1; υ is a positive constant; and $\varepsilon ={\left[\begin{array}{cc} \varepsilon_{1} & \varepsilon_{2} \end{array}\right]}^{T}$ is small and positive, which satisfies $\left(1/\varpi b_{3}-1\right)\left|{\bar{u}}_{eq}(t)\right|+\varepsilon /2>0$.

In Equation (34), the equivalent control variable ${\bar{u}}_{eq}(t)$ is based on a first-order low pass filter, to filter the discontinuous injection signal σ, and the error variable δ(t) is designed to satisfy δ(t)→0, as t→0. Hence, to improve the filtering performance and the convergent speed of the error variable, a novel equivalent control variable ${\bar{u}}_{eq}(t)$ and its error variable δ(t) are formulated as

(35) $$\begin{array}{l} \bar{\delta }(t)=L\left(t\right)-\frac{1}{\varpi }\left|{\bar{u}}_{eq}(t)\right|-\varepsilon \\ \delta (t)={\left|\bar{\delta }(t)\right|}^{\kappa }\mathrm{sign}\left(\bar{\delta }(t)\right)\\ {\ddot{\bar{u}}}_{eq}(t)=\frac{1}{\upsilon_{1}}\left(a_{3}(t)\circ \frac{\sigma }{\left\|\sigma \right\|}+a_{4}(t)\circ \sigma -\upsilon_{2}{\dot{\bar{u}}}_{eq}(t)-{\bar{u}}_{eq}(t)\right) \end{array}$$

where $\upsilon_{1}$ and $\upsilon_{2}$ are positive constants, and 0<κ<1. Compared with Equation (34), the error variable δ(t) in Equation (35) has a fast convergence form which can drive the value of the adaptive gain to counteract the equivalent control variable of disturbance ${\bar{u}}_{eq}(t)$ in faster speed.

Then, the adaptive time-varying gain L(t) is given by

(36) $$\begin{array}{l} L(t)=l_{0}+l(t)\\ \dot{l}(t)=-\rho (t)\circ \delta (t)\\ \rho (t)=q_{0}+q(t)\\ \dot{q}(t)=\varsigma \left|\delta (t)\right| \end{array}$$

where $l_{0}={\left[\begin{array}{cc} l_{01} & l_{02} \end{array}\right]}^{T}$ is the initial value of l(t); $q_{0}={\left[\begin{array}{cc} q_{01} & q_{02} \end{array}\right]}^{T}$ is the initial value of q(t); and ς is a positive constant.

**Proposition** **1.** *Following Equations (34)–(36), the STA adaptive gain L(t) satisfies $L(t)\geq \left|\dot{d}(t)\right|$ in finite time.*

**Proof of Proposition** **1.** See Appendix B. □

### 4.2. Stability Analysis of The Proposed Guidance Law

The main conclusion of this part is summarized as following theorem.

**Theorem** **1.** *Consider the three-dimension missile-target engagement system with terminal angle constraint (Equation (6)), the proposed adaptive STA guidance law (Equation (32)) can drive the sliding surface converge to a small neighborhood around zero in finite time $T_{reach}$, if the adaptive gain factors are governed as*

(37) $$9b_{1}^{2}b_{2}^{2}+8b_{2}^{2}b_{3}-4b_{3}b_{4}<0$$

*and $T_{reach}$ is given by*

(38) $$T_{r}\leq \frac{2}{\zeta_{1}}\ln\left(1+\frac{\zeta_{1}}{\zeta_{2}}V^{1/2}(x_{0})\right)$$

*where $\zeta_{1}$ and $\zeta_{2}$ are denoted in Equation (58).*

**Proof of Theorem** **1.** Substituting Equation (32) into Equation (30) and replacing y(t) by $\bar{y}(t)$ yields

(39) $$\begin{array}{l} \dot{\sigma }={\left[\begin{array}{cc} {\dot{\sigma }}_{1} & {\dot{\sigma }}_{2} \end{array}\right]}^{T}=-a_{1}(t)\circ \frac{\sigma }{{\left\|\sigma \right\|}^{1/2}}-a_{2}(t)\circ \sigma +\bar{y}(t)\\ \dot{\bar{y}}(t)={\left[\begin{array}{cc} {\dot{\bar{y}}}_{1}(t) & {\dot{\bar{y}}}_{2}(t) \end{array}\right]}^{T}=-a_{3}(t)\circ \frac{\sigma }{\left\|\sigma \right\|}-a_{4}(t)\circ \sigma +\dot{\Delta } \end{array}$$

In order to simplify the Hadamard product operations, consider the adaptive gain L(t) as a scalar quantity form L(t), and Equation (39) can be rewritten as

(40) $$\begin{array}{l} \dot{\sigma }=-a_{1}(t)\frac{\sigma }{{\left\|\sigma \right\|}^{1/2}}-a_{2}(t)\sigma +\bar{y}(t)\\ \dot{\bar{y}}(t)=-a_{3}(t)\frac{\sigma }{\left\|\sigma \right\|}-a_{4}(t)\sigma +\dot{\Delta } \end{array}$$

Introduce an auxiliary vector z, which is formulated as

(41) $$z=\left[\begin{array}{c} z_{1}\\ z_{2}\\ z_{3} \end{array}\right]=\left[\begin{array}{c} \sqrt{L}\sigma /{\left\|\sigma \right\|}^{1/2}\\ L\sigma \\ \bar{y} \end{array}\right]$$

Then, the derivative of z is given by

(42) $$\dot{z}=\left[\begin{array}{c} {\dot{z}}_{1}\\ {\dot{z}}_{2}\\ {\dot{z}}_{3} \end{array}\right]=\left[\begin{array}{c} \frac{\dot{L}\sigma }{2{\left(L\left\|\sigma \right\|\right)}^{1/2}}+{\left(\frac{L}{\left\|\sigma \right\|}\right)}^{1/2}\left(I_{2\times 2}-\frac{\sigma \sigma^{T}}{2{\left\|\sigma \right\|}^{2}}\right)\dot{\sigma }\\ \dot{L}\sigma +L\dot{\sigma }\\ -\frac{\gamma (t)}{\left\|\sigma \right\|}\sigma +\dot{\Delta } \end{array}\right]$$

Consider following Lyapunov function candidate

(43) $$V=\frac{1}{2}z^{T}Pz$$

where

(44) $$P=\left[\begin{array}{ccc} \left(4b_{3}+b_{1}^{2}\right)I_{2\times 2} & b_{1}b_{2}I_{2\times 2} & -b_{1}I_{2\times 2}\\ b_{1}b_{2}I_{2\times 2} & (2b_{4}+b_{2}^{2})I_{2\times 2} & -b_{2}I_{2\times 2}\\ -b_{1}I_{2\times 2} & -b_{2}I_{2\times 2} & 2I_{2\times 2} \end{array}\right]$$

From Equation (43), one can conclude that

(45) $$\lambda_{\min}(P){\left\|z\right\|}^{2}\leq z^{T}Pz\leq \lambda_{\max}(P){\left\|z\right\|}^{2}$$

Taking the derivative of V into time yields

(46) $$\dot{V}=\frac{L}{4\left\|z_{1}\right\|}z^{T}\left(A_{1}^{T}P+PA_{1}\right)z-\frac{Lz^{T}}{2}\left(A_{2}^{T}P+PA_{2}\right)z+z^{T}PA_{3}$$

where

$$\begin{array}{l} A_{1}=\left[\begin{array}{ccc} b_{1}I_{2\times 2} & b_{2}I_{2\times 2} & -2I_{2\times 2}\\ 0_{2\times 2} & 0_{2\times 2} & 0_{2\times 2}\\ 2b_{3}I_{2\times 2} & 0_{2\times 2} & 0_{2\times 2} \end{array}\right]\;A_{2}=\left[\begin{array}{ccc} 0_{2\times 2} & 0_{2\times 2} & 0_{2\times 2}\\ b_{1}I_{2\times 2} & b_{2}I_{2\times 2} & -I_{2\times 2}\\ 0_{2\times 2} & b_{4}0_{2\times 2} & 0_{2\times 2} \end{array}\right]\\ A_{3}=A_{31}+A_{32}+A_{33}=\frac{\dot{L}}{2L}\left[\begin{array}{c} z_{1}\\ 2z_{2}\\ 0_{2} \end{array}\right]+\left[\begin{array}{c} 0_{2}\\ 0_{2}\\ \dot{\Delta } \end{array}\right]+\left[\begin{array}{c} -Lz_{1}z_{1}^{T}z_{3}/\left(2{\left\|z_{1}\right\|}^{3}\right)\\ 0_{2}\\ 0_{2} \end{array}\right] \end{array}$$

Denote $\tilde{V}=z^{T}PA_{3}$ and ${\tilde{V}}_{i}=z^{T}PA_{3i},i=1,2,3$. Thus, $\tilde{V}$ can be written as

(47) $$\tilde{V}={\tilde{V}}_{1}+{\tilde{V}}_{2}+{\tilde{V}}_{3}$$

where

(48) $${\tilde{V}}_{1}=\frac{\dot{L}}{2L}[\left(4b_{3}+b_{1}^{2}\right){\left\|z_{1}\right\|}^{2}+3b_{1}b_{2}z_{1}^{T}z_{2}-b_{1}z_{1}^{T}z_{3}+2\left(2b_{4}+b_{2}^{2}\right){\left\|z_{2}\right\|}^{2}-2b_{2}z_{2}^{T}z_{3}]$$

(49) $${\tilde{V}}_{2}={\dot{\Delta }}^{T}\left[2z_{3}-b_{1}z_{1}-b_{2}z_{2}\right]$$

(50) $${\tilde{V}}_{3}=-\frac{L}{2\left\|z_{1}\right\|}\left[\left(4b_{3}+b_{1}^{2}\right)z_{1}^{T}z_{3}+\frac{b_{1}b_{2}z_{2}^{T}z_{1}z_{1}^{T}z_{3}}{{\left\|z_{1}\right\|}^{2}}-\frac{b_{1}z_{3}^{T}z_{1}z_{1}^{T}z_{3}}{{\left\|z_{1}\right\|}^{2}}\right]$$

It can be concluded that Equations (48)–(50) satisfy

(51) $${\tilde{V}}_{1}\leq \frac{\dot{L}}{2L}z^{T}\Omega z$$

(52) $${\tilde{V}}_{2}\leq \sqrt{4+b_{1}^{2}+b_{2}^{2}}\left\|\dot{\Delta }\right\|\left\|z\right\|$$

(53) $${\tilde{V}}_{3}\leq -\frac{L}{2\left\|z_{1}\right\|}\left[\left(4b_{3}+b_{1}^{2}\right)z_{1}^{T}z_{3}-b_{1}{\left\|z_{3}\right\|}^{2}\right]-\frac{Lb_{1}b_{2}}{2}z_{1}^{T}z_{3}$$

where

$$\begin{array}{l} \Omega_{1}=diag\left(\Omega_{10},\Omega_{20},\Omega_{30}\right)⊗I_{2\times 2}\\ \Omega_{10}=4b_{3}+b_{1}^{2}+1.5b_{1}b_{2}+0.5b_{1}\\ \Omega_{20}=4b_{4}+2b_{2}^{2}+1.5b_{1}b_{2}+b_{2}\\ \Omega_{30}=0.5b_{1}+b_{2} \end{array}$$

Then, the derivative of the Lyapunov function satisfy the following inequality

(54) $$\dot{V}\leq -\frac{L}{2\left\|z_{1}\right\|}z^{T}\Omega_{2}z-Lz^{T}\Omega_{3}z+{\tilde{V}}_{1}+{\tilde{V}}_{2}$$

where

$$\begin{array}{c} \Omega_{2}=\left[\begin{array}{ccc} b_{1}(b_{1}^{2}+2b_{3}) & 0 & -b_{1}^{2}\\ 0 & b_{1}(5b_{2}^{2}+2b_{4}) & -3b_{1}b_{2}\\ -b_{1}^{2} & -3b_{1}b_{2} & b_{1} \end{array}\right]⊗I_{2\times 2}\\ \Omega_{3}=\left[\begin{array}{ccc} b_{2}(2b_{1}^{2}+b_{3}) & 0 & 0\\ 0 & b_{2}(b_{2}^{2}+b_{4}) & -b_{2}^{2}\\ 0 & -b_{2}^{2} & b_{2} \end{array}\right]⊗I_{2\times 2} \end{array}$$

It can be concluded that $\Omega_{2}$ and $\Omega_{3}$ are positive definite matrices under condition (37). Thus, $\Omega_{i},i=1,2,3$ satisfy

(55) $$\lambda_{\min}(\Omega_{i}){\left\|z\right\|}^{2}\leq z^{T}\Omega_{i}z\leq \lambda_{\max}(\Omega_{i}){\left\|z\right\|}^{2},i=1,2,3$$

Considering (45) and (55), the following condition holds

(56) $$\frac{\lambda_{\min}(\Omega_{i})V}{\lambda_{\max}(P)}\leq z^{T}\Omega_{i}z\leq \frac{\lambda_{\max}(\Omega_{i})}{\lambda_{\min}(P)},i=1,2,3$$

Substituting (51), (52) and (56) into (54) yields

(57) $$\begin{array}{ll} \dot{V} & \leq \left[\frac{\dot{L}\lambda_{\max}(\Omega_{1})}{L\lambda_{\min}(P)}-\frac{2L\lambda_{\min}(\Omega_{3})}{\lambda_{\max}(P)}\right]V-\frac{L\lambda_{\min}(\Omega_{2})}{\left\|z_{1}\right\|\lambda_{\max}(P)}V+\sqrt{\left(4+b_{1}^{2}+b_{2}^{2}\right)}\left\|z\right\|{\dot{\Delta }}_{\max}\\ & \leq -\left(L\gamma_{2}-\frac{\dot{L}}{L}\gamma_{1}\right)V-\left(L\gamma_{3}-\gamma_{4}{\dot{\Delta }}_{\max}\right)V^{1/2} \end{array}$$

where

$$\gamma_{1}=\frac{\lambda_{\max}(\Omega_{1})}{\lambda_{\min}(P)},\;\gamma_{2}=\frac{2\lambda_{\min}(\Omega_{3})}{\lambda_{\max}(P)},\;\gamma_{3}=\frac{\lambda_{\min}(\Omega_{2})\sqrt{\lambda_{\min}(P)}}{\sqrt{2}\lambda_{\max}(P)},\;\gamma_{4}=\sqrt{\frac{2(4+b_{1}^{2}+b_{2}^{2})}{\lambda_{\min}(P)}}$$

Letting

(58) $$\zeta_{1}=L\gamma_{2}-\dot{L}\gamma_{1}/L,\;\zeta_{2}=L\gamma_{3}-\gamma_{4}{\dot{\Delta }}_{\max}$$

It can be found that $\zeta_{1}$ and $\zeta_{2}$ are positive in finite time under Proposition 1. And based on Lemma 1, the proposed guidance law is stabilized in finite time. □

## 5. Simulation

In this section, several simulation situations are designed to study the effectiveness of the proposed measurement compensation system and guidance law. First, the effectiveness of the compensation system based on predictive ADRC is discussed; next, variable desired terminal angles are taken in consideration to test the performance of the proposed guidance law with the compensation system. Finally, compared with other guidance laws, the property of the proposed guidance law with measurement compensation system is demonstrated further. All the simulations are supported by MATLAB platform due to its adequate libraries and powerful matrix calculation ability. Throughout the simulations, a fourth-order Runge-Kutta solver with a fixed step size is used.

### 5.1. Simulation of The Predicrive ADRC Compensation System

A simulation example of missile-target engagement guidance is investigated to verify the effectiveness of the proposed predictive ADRC compensation system (Equations (15)–(23)). In addition, this example is simulated on the basis of the proposed guidance law (Equation (32)).

Since the capacity of actuator dynamics is limited in real practice, the maximum lateral acceleration $a_{M}$ is limited as follows

(59) $$a_{M}=\{\begin{array}{cc} a_{M,\max}sign\left(a_{M}\right) & if\left|a_{M}\right|\geq a_{M,\max}\\ a_{M} & if\left|a_{M}\right|<a_{M,\max} \end{array}$$

This paper selects $a_{M,\max}=300{\;m/s}^{2}$ throughout the simulations.

To address the discontinuity problem of the sign function *sign*(*x*), a sigmoid function is utilized to replace it during the simulation, which is formulated as

(60) $$sigmoid(x)=\frac{2}{1+e^{-x}}-1$$

The simulation example is set up as follows: (1) the initial missile-target engagement condition: $r_{0}=8000\;m$, ${\dot{r}}_{0}=-800\;m/s$, $\theta_{0}=15^{\circ }$, $\phi_{0}=45^{\circ }$, ${\dot{\theta }}_{0}={\dot{\phi }}_{0}=0^{\circ }/s$, $\theta_{d}=40^{\circ }$ and $\phi_{d}=60^{\circ }$; (2) the target maneuver condition: $a_{T\theta }=a_{T\phi }=-30\sin(0.4\pi t)\;{m/s}^{2}$, and $a_{Tr}=0\;{m/s}^{2}$; (3) the seeker parameters: $T_{s}=0.2\;s$, τ=0.1 s, output noise is zero-mean Gaussian white noise with power spectral density $S_{w}=0.0001\;W/\mathrm{Hz}$, and its variance is 0.0001 °/s considering narrow band; (4) the parameters of measurement compensation system are given in Table 1; (5) the parameters of sliding mode manifold and adaptive STA are given in Table 2; and (6) the fixed step size of the fourth-order Runge-Kutta solver is 0.001 s.

**Figure 4:**
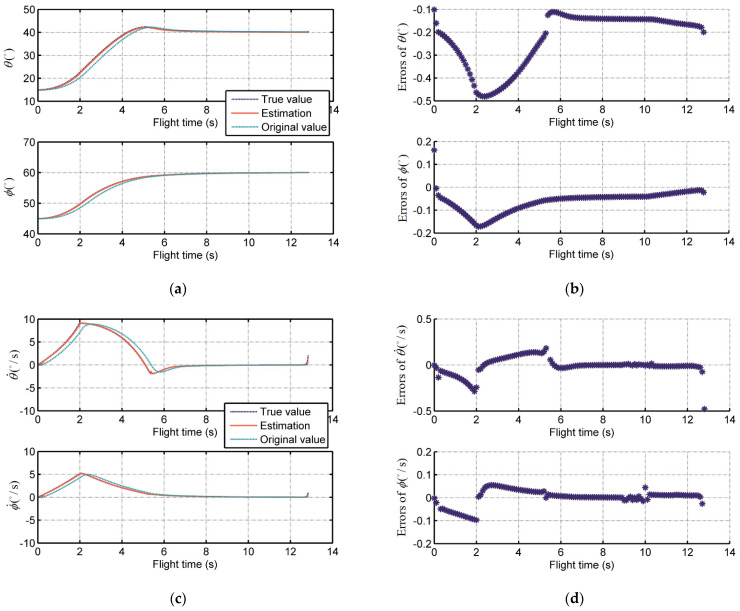
The performance of the proposed predictive ADRC compensation system for seeker lag and time delay: (**a**) True value, estimation, and original value of LOS angle; (**b**) Errors between true value and estimation of LOS angle; (**c**) True value, estimation, and original value of LOS angular rate; (**d**) Errors between true value and estimation of LOS angular rate.

The simulation results are illustrated in Figure 4. Figure 4a,c show the LOS angle and LOS angular rate estimations of predictive ADRC compensation system, respectively. The estimation could track the true value calculated by the missile-target engagement dynamics in the overall guidance process. The original value detected by the time-delay seeker is also given as a comparison. Moreover, the errors between them are shown in Figure 4b,d, which illustrates the good performance of the predictive ADRC compensation system quantitively. The error ranges of LOS angles θ and ϕ are (−0.5° to −0.1°) and (−0.2° to −0.2°), respectively, and that of LOS angular rates $\dot{\theta }$ and $\dot{\phi }$ are (−0.4 °/s to 0.2 °/s) and (−0.1 °/s to −0.1 °/s), respectively.

**Figure 5:**
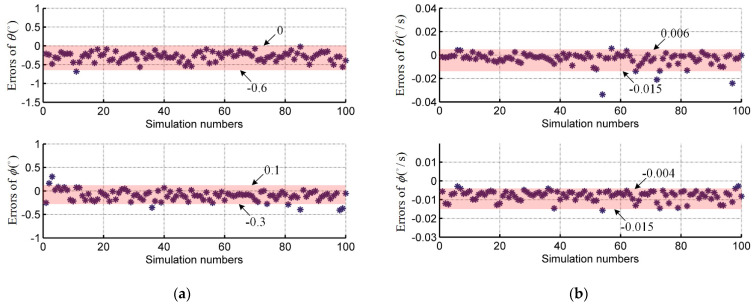
The Monte Carlo simulation result of the errors: (**a**) The LOS angle errors in 100-times simulation; (**b**) The LOS angular rate errors in 100-times simulation.

Figure 5 shows the Monte Carlo simulation result, where the single scatter point represents the average value of the errors between estimation values and true values during the entire flight time in one Monte Carlo run. Excluding a few large error scatters, the range of the error mean value can be governed. The mean value error ranges of LOS angles θ and ϕ are (−0.6° to 0°) and (−0.3° to 0.1°), respectively, and that of LOS angular rates $\dot{\theta }$ and $\dot{\phi }$ are (−0.015 °/s to 0.006 °/s) and (−0.015 °/s to −0.004 °/s), respectively. Those small errors demonstrate the strong property of robustness of the proposed predictive ADRC compensation system.

### 5.2. Simulation of Different Terminal Angle Constraints

In order to test the terminal angle constraint properties of the proposed guidance law with the ADRC predictor observer, a simulation example of missile-target engagement guidance is investigated. The missile is expected to hit the target in the desired terminal angles: $\phi_{d}$ = 70°, 60°, 50°, 30°, and 20°, when $\theta_{d}$ = 45°; $\theta_{d}$ = 45°, 30°, 20°, 0°, and −15°, when $\phi_{d}$ = 15°. Other initial conditions and system parameters are the same as in Section 5.1.

**Figure 6:**
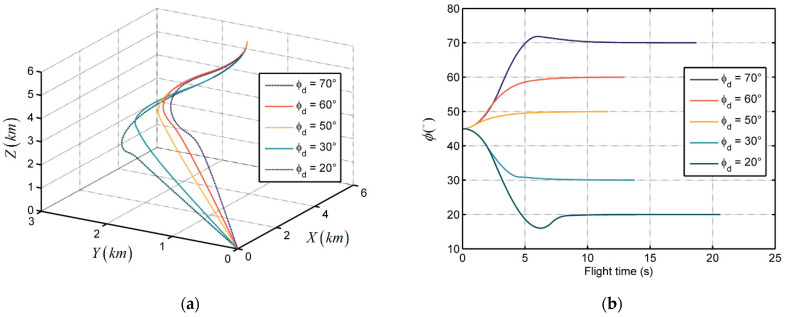
The performance of the proposed guidance law with different desired terminal azimuth angles: (**a**) Relative distance trajectories; and (**b**) LOS angles.

**Figure 7:**
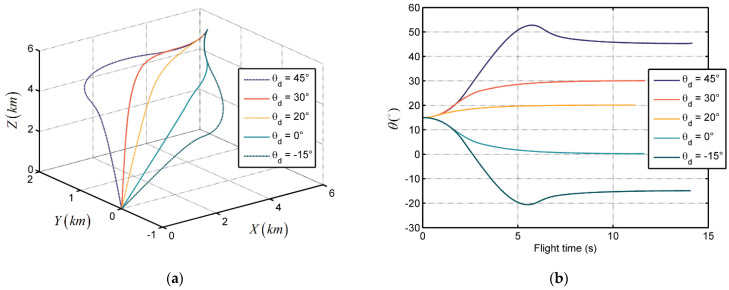
The performance of the proposed guidance law with different desired terminal elevation angles: (**a**) Relative distance trajectories; and (**b**) LOS angles.

The simulation results are illustrated in Figure 6 and Figure 7. Figure 6a and Figure 7a show the trajectories of missile target relative distances in the inertial coordinate system when $\theta_{d}$ = 45° and $\phi_{d}$ = 15°. We see that all the trajectories converge to zero, which implies that the missile can catch up with the target in finite time without obtaining target acceleration information. Figure 6b and Figure 7b show the trend of LOS angle over flight time in the two conditions. In this sample, the LOS angles θ and ϕ can reach the desired terminal angle within 7 s and 10 s, respectively, and then maintain it. This phenomenon verifies the terminal angle and seeker delay constraint properties of the proposed guidance law. Moreover, the convergence time and flight time are subject to the difference between the initial LOS angle and desired terminal angle. Larger gaps could lead to longer trajectories and longer convergence time.

### 5.3. Compared with Other Guidance Laws

The fixed-gain STA-based sliding mode control (STASMC) guidance law and fast convergent terminal sliding mode (TSM) guidance law are introduced for comparison. The fixed-gain STASMC guidance law is formulated as [35]

(61) $$\begin{array}{l} a_{M}=-B^{-1}\left(\bar{F}(x)+k_{1}x_{2}+k_{2}\dot{w}(x_{1})+c_{1}\frac{\sigma }{{\left\|\sigma \right\|}^{1/2}}-c_{2}\sigma +\Phi (t)\right)\\ \dot{\Phi }(t)=-c_{3}\frac{\sigma }{\left\|\sigma \right\|}-c_{4}\sigma \end{array}$$

This paper selects the parameters of (62) as $c_{1}=1.6$, $c_{2}=0.1$, $c_{3}=0.4$, and $c_{4}=0.1$.

The TSM guidance law is given by [28]

(62) $$\begin{array}{l} a_{M}=-B^{-1}\left(\bar{F}(x)-Ba_{T}+k_{1}x_{2}+k_{2}\dot{w}(x_{1})+v_{1}{\left|\sigma \right|}^{1-1/v_{2}}\mathrm{sign}\left(\sigma \right)-v_{3}\bar{\Phi }\right)\\ \dot{\bar{\Phi }}(t)=B{\left|\sigma \right|}^{1-2/v_{2}}\mathrm{sign}\left(\sigma \right) \end{array}$$

This paper selects the parameters of (62) as $v_{1}=5$, $v_{2}=0.8$, and $v_{3}=1$, and assumes the target acceleration $a_{T}$ is known for ease.

To further verify the applicability and robustness of the proposed guidance law and the proposed predictive ADRC compensation system, three kinds of scenarios with various measurement and target maneuvers are taken into account:

1. The seeker measurement is ideal, which has no delays or noises. The target accelerations in LOS coordinate is given by $a_{T\theta }=a_{T\phi }=-30\sin(0.4\pi t)\;{m/s}^{2}$;
2. The seeker measurement is ideal, which has no delays or noises. The target accelerations in LOS coordinate is given by $a_{T\theta }=a_{T\phi }=-30\;{m/s}^{2}$;
3. The seeker measurement has delays and noises, which are given by $T_{s}=0.2\;s$, τ=0.1 s, $S_{w}=0.0001\;W/\mathrm{Hz}$. The target accelerations in LOS coordinate is given by $a_{T\theta }=a_{T\phi }=-30\sin(0.4\pi t)\;{m/s}^{2}$.

Besides the above, the initial conditions and parameters of these three scenarios are as same as that in Section 5.1.

**Figure 8:**
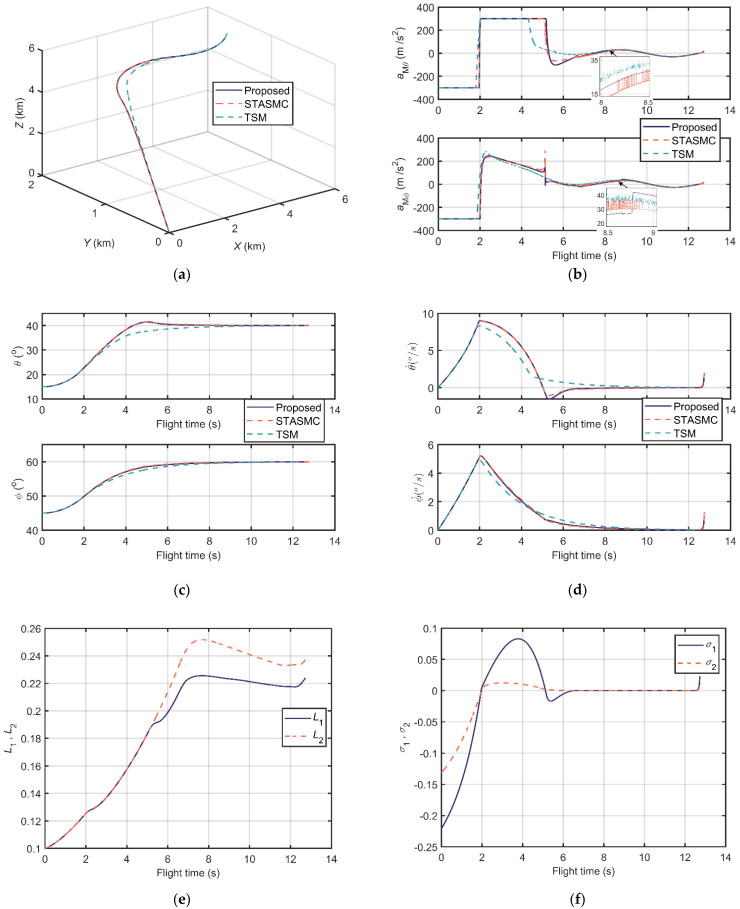
Comparison results of scenario 1: (**a**) Relative distance trajectories; (**b**) Output accelerations of guidance loops; (**c**) LOS angles; (**d**) LOS angular rates; (**e**) The adaptive STA gains; and (**f**) The sliding manifold.

**Figure 9:**
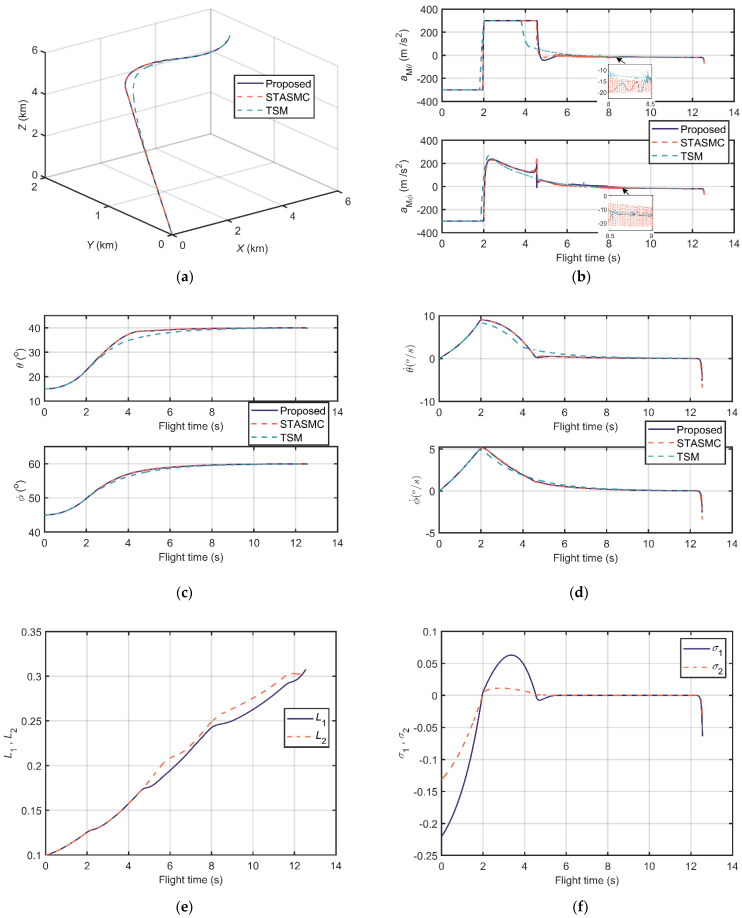
Comparison results of scenario 2: (**a**) Relative distance trajectories; (**b**) Output accelerations of guidance loops; (**c**) LOS angles; (**d**) LOS angular rates; (**e**) The adaptive STA gains; (**f**) The sliding manifold.

The comparison results of scenarios 1 and 2 are shown in Figure 8 and Figure 9, respectively. Figure 8a and Figure 9a illustrate the relative distance trajectories, and all the three guidance laws converge to zero. In Figure 8b and Figure 9b, one can observe that the acceleration output of the proposed guidance law is continuous and smooth. Contrarily, the accelerations of STASMC and TSM chatter significantly. The LOS angle curves in Figure 8c and Figure 9c and the LOS angular rate curves in Figure 8d and Figure 9d depict the terminal angle constrained property of the guidance laws. The LOS angles of these three conditions can reach the desired terminal angle and the LOS angle rates can converge to zero. Figure 8e and Figure 9e show the trend of the adaptive STA gains of the proposed guidance law, which are bounded during flight time. The sliding variables of the proposed scenarios reach zero at approximately 6 s and 5 s, respectively, as shown in Figure 8f and Figure 9f.

Moreover, other information including flight time, miss distance, settling time (±0.5°), and error of LOS angles is listed in Table 3 and Table 4. Miss distance states the distance between missile and target at the end. Settling time (±0.5°) indicates the time that LOS angles reach the ±0.5° neighborhood of the desired terminal angles and then maintain it. Error of *θ* and error of *ϕ* are errors between simulation terminal angles and desired terminal angles. From Table 3 and Table 4, one can conclude that the proposed guidance law has a superior performance in miss distance, settling time (±0.5°), and terminal LOS angle errors in these two designed scenarios.

**Figure 10:**
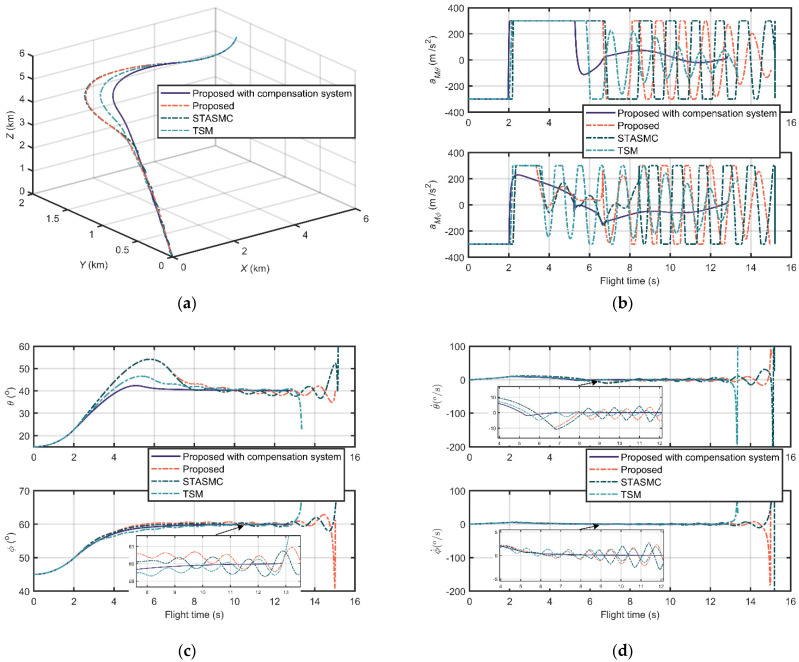
Comparison results of scenario 3: (**a**) Relative distance trajectories; (**b**) Output accelerations of guidance loops; (**c**) LOS angles; and (**d**) LOS angular rates.

Unlike Figure 8 and Figure 9, the comparison results of scenario 3 in Figure 10 take the proposed compensation system in account as well. Figure 10a is the relative distance trajectories of the proposed guidance law with the proposed compensation system and the three guidance laws without measurement compensation structures. It is obvious that the trajectories of those without compensation have distinct chattering, and are longer than those proposed with a compensation system. The chattering also occurs in the aspects of output accelerations, LOS angles, and LOS angular rates, as shown in Figure 10b, Figure 10c and Figure 10d, respectively. The chattering phenomenon is caused by the additional seeker lag, measurement delay, and noise. Due to this reason, the LOS angle and LOS angular rate curves of these three guidance laws do not converge. On the other hand, due to the compensation property of the proposed compensation system for lags, delays, and noises, the curves of the proposed guidance law with compensation system are smooth and stable in Figure 10b–d.

Similarly, the information about flight time, miss distance, settling time (±0.5°), and LOS angle error is listed in Table 5. We can observe that the proposed guidance law with compensation has the best performance in all aspects in this scenario. It should be noted that the settling time and LOS angle error of the proposed guidance law, STASMC, and TSM are not available due to their divergent LOS angles. Moreover, compared with the property of the proposed of scenario 1 listed in Table 3, the proposed with compensation maintains broadly stable flight times and miss distances, but have a slightly decreasing performance in settling time and LOS angle error.

As a result, compared with STASMC and TSM, the proposed guidance law has no output chattering, less terminal LOS angle settling time, and error. In addition, the system which consists of the proposed guidance law and the proposed ADRC compensation structure has excellent seeker lag, delay, and noise constraint property and robustness.

## 6. Conclusions

In order to address the lag and delay problems of the seeker measurement, this paper presents a novel measurement compensation system for tactical missiles. Moreover, this paper presents a novel adaptive guidance law regardless of target maneuvers which has fast convergent gains. The simulation results demonstrate the strong disturbance rejection and accuracy property of the proposed compensation system. Also, the simulations verify the effectiveness and robustness of the proposed guidance law.

Specifically, the details of the proposed method can be summarized as follows:

1. The measurement compensation system has a predictive ADRC structure, which consists of a predictive tracking loop and an ADRC loop. The predictive tracking loop could follow a virtual measurement without lags and delays. The ADRC loop ensures the tracking process stable under disturbances;
2. The sliding mode guidance law is based on adaptive STA control. In order to speed the convergence of the gain error, a fast convergent error form is proposed implementing the equivalent control method to design adaptive gains. In addition, the control system is proofed for finite time convergence.

## Figures and Tables

**Table 1:** The parameters of measurement compensation system.

Parameter	Value	Parameter	Value
$p_{0}$	0.5	$\beta_{1}$	0.21
$h_{0}$	0.001	$\beta_{2}$	2.23
α	0.4	$\beta_{3}$	1.47
$\bar{\tau }$	0.5	μ	0.1
R	5	δ	0.1

**Table 2:** The parameters of sliding mode manifold and adaptive STA.

Parameter	Value	Parameter	Value
ξ	0.2	$b_{1}$	5
$k_{1}$	0.5	$b_{2}$	0.1
$k_{2}$	0.1	$b_{3}$	1.2
$k_{3}$	2.5	$b_{4}$	1
$\varepsilon_{1},\varepsilon_{2}$	0.2	κ	0.5
ς	0.1	ϖ	0.67
$\upsilon_{1}$	0.5	$\upsilon_{2}$	0.2
$l_{01},l_{02}$	0.1	$q_{01},q_{02}$	0.1

**Table 3:** Simulation results of scenario 1.

Guidance Law	Flight Time	Miss Distance	Settling Time of *θ* (±0.5°)	Settling Time of *ϕ* (±0.5°)	Error of *θ*	Error of *ϕ*
Proposed	12.745 s	0.0218 m	5.923 s	6.733 s	0.0187°	0.0057°
STASMC	12.756 s	0.3915 m	6.460 s	7.057 s	0.0532°	0.0369°
TSM	12.204 s	0.1018 m	8.084 s	8.025 s	0.0635°	0.0617°

**Table 4:** Simulation results of scenario 2.

Guidance Law	Flight Time	Miss Distance	Settling Time of *θ* (±0.5°)	Settling Time of *ϕ* (±0.5°)	Error of *θ*	Error of *ϕ*
Proposed	12.567 s	0.0108 m	6.783 s	7.391 s	0.0350°	0.0450°
STASMC	12.572 s	0.1043 m	6.908 s	7.488 s	0.0430°	0.0510°
TSM	12.141 s	0.1009 m	8.692 s	9.032 s	0.0891°	0.0643°

**Table 5:** Simulation results of scenario 3.

Guidance Law	Flight Time	Miss Distance	Settling Timeof *θ* (±0.5°)	Settling Timeof *ϕ* (±0.5°)	Error of *θ*	Error of *ϕ*
Proposed with compensation	12.857 s	0.0163 m	7.687 s	6.825 s	0.1993°	0.0058°
Proposed	15.024 s	0.3282 m	None	None	None	None
STASMC	15.194 s	2.2072 m	None	None	None	None
TSM	13.362 s	0.3880 m	None	None	None	None

## Data Availability

The data presented in this study are available on request from the corresponding author.

## Appendix A

**Table A1:** The list of acronyms used in the paper.

Acronym	Full Name
ADRC	active disturbance rejection control
SMC	sliding mode control
LOS	Line-of-sight
STA	super-twisting algorithm
TD	tracking differentiator
SF	state feedback
SEF	state error feedback
ESO	extended state observer
STASMC	STA-based sliding mode control
TSM	terminal sliding mode

## Appendix B

**Proof of Proposition** **1.** Letting $\iota (t)=\dot{d}(t)$, and satisfying $\left\|\iota (t)\right\|\leq d_{1}$ and $\left\|\dot{\iota }(t)\right\|\leq d_{2}$. Defining an auxiliary variable as

(A1) $$\dot{\bar{\delta }}(t)=\dot{l}(t)-\frac{1}{\varpi }\left|{\dot{\bar{u}}}_{eq}(t)\right|$$

Taking the derivative of $\bar{\delta }(t)$ to time yields

(A2) $$\dot{\bar{\delta }}(t)=\dot{l}(t)-\frac{1}{\varpi }\left|{\dot{\bar{u}}}_{eq}(t)\right|$$

Then,

(A3) $$\begin{array}{ll} \bar{\delta }(t)\circ \dot{\bar{\delta }}(t) & \leq \bar{\delta }(t)\circ \dot{l}(t)+\frac{d_{1}}{\varpi }\dot{\bar{\delta }}(t)\\ & =-q_{0}\circ \left|\bar{\delta }(t)\right|-q\circ \left|\bar{\delta }(t)\right|+\frac{d_{1}}{\varpi }\dot{\bar{\delta }}(t)\\ & =-q_{0}\circ \left|\bar{\delta }(t)\right|+e(t)\circ \left|\bar{\delta }(t)\right| \end{array}$$

Take following Lyapunov function

(A4) $$V=\frac{1}{2}\bar{\delta }\circ \bar{\delta }+\frac{1}{2\varsigma }e\circ e$$

Thus, the derivative of V is formulated as

(A5) $$\begin{array}{ll} \dot{V} & =\bar{\delta }\circ \dot{\bar{\delta }}+\frac{1}{\varsigma }e\circ \dot{e}\\ & \leq -q_{0}\circ \left|\bar{\delta }\right|+e\circ \left|\bar{\delta }\right|+\frac{1}{\varsigma }e\circ (-\varsigma \left|\bar{\delta }\right|)\\ & =-q_{0}\circ \left|\bar{\delta }\right|\leq 0 \end{array}$$

One can observe that $\bar{\delta }(t)$ is bounded, and when t→∞, $\bar{\delta }(t)\to 0$. Hence, there exists a finite time $t_{0}$ such that $\left|\bar{\delta }(t)\right|\leq \varepsilon /2$ holds for $t>t_{0}$, which can be written as

(A6) $$\left|\bar{\delta }(t)\right|=\left|L\left(t\right)-\frac{1}{\varpi }\left|{\bar{u}}_{eq}(t)\right|-\varepsilon \right|\leq \frac{\varepsilon }{2}$$

If ϖ<1, L(t) satisfies

(A7) $$L\left(t\right)\geq \frac{1}{\varpi }\left|{\bar{u}}_{eq}(t)\right|+\frac{\varepsilon }{2}\geq \left|{\bar{u}}_{eq}(t)\right|+\frac{\varepsilon }{2}\geq \left|\dot{d}(t)\right|$$

This completes the proof. □
